# Understanding the Heterogeneity of Obesity and the Relationship to the Brain-Gut Axis

**DOI:** 10.3390/nu12123701

**Published:** 2020-11-30

**Authors:** Tony K. W. Hung, Tien S. Dong, Zixi Chen, David Elashoff, Janet S. Sinsheimer, Jonathan P. Jacobs, Venu Lagishetty, Priten Vora, Jean Stains, Emeran A. Mayer, Arpana Gupta

**Affiliations:** 1 Division of Hematology and Oncology, University of California, Los Angeles, CA 90095, USA; TonyHung@ucla.edu (T.K.W.H.); TSDong@mednet.ucla.edu (T.S.D.); Chelsea991205@outlook.com (Z.C.); DElashoff@mednet.ucla.edu (D.E.); JJacobs@mednet.ucla.edu (J.P.J.); VLagishetty@mednet.ucla.edu (V.L.); PPVora@mednet.ucla.edu (P.V.); JStains@mednet.ucla.edu (J.S.); EMayer@mednet.ucla.edu (E.A.M.); 2 David Geffen School of Medicine, UCLA, Los Angeles, CA 90095, USA; 3 Vatche and Tamar Manoukian Division of Digestive Diseases, UCLA, Los Angeles, CA 90095, USA; 4 UCLA Microbiome Center, Los Angeles, CA 90095, USA; 5 Division of Gastroenterology, Hepatology and Parenteral Nutrition, Veterans Administration Greater Los Angeles Healthcare System, Los Angeles, CA 90095, USA; 6 G. Oppenheimer Center for Neurobiology of Stress and Resilience, UCLA, Los Angeles, CA 90095, USA; 7 Department of Computational Medicine, David Geffen School of Medicine at UCLA, Los Angeles, CA 90095, USA; jsinshei@ucla.edu; 8 Department of Biostatistics, UCLA Fielding School of Public Health, Los Angeles, CA 90095, USA; 9 Department of Human Genetics, David Geffen School of Medicine at UCLA, Los Angeles, CA 90095, USA; 10 Ahmanson-Lovelace Brain Mapping Center, UCLA, Los Angeles, CA 90095, USA

**Keywords:** obesity, microbiome, brain-gut axis, amino acids, metabolites, heterogeneity, precision medicine, Hispanic, health disparity, diet

## Abstract

Obesity is best understood as a multifactorial metabolic imbalances disorder. In a cross-sectional study, we aimed to explore sociodemographic and dietary determinants of obesity in relation to brain-gut homeostasis among overweight and obese individuals. Multivariate logistic regression models were used to examine obesity and its association with sociodemographic and dietary factors. Biological variables examined included the gut microbiome, fecal amino acid metabolites and brain structural volumes. Among 130 participants, there were higher odds of obesity if individuals were Hispanic (adjusted odds ratio (aOR) 1.56, *p* = 0.014). Compared to non-Hispanics, Hispanics differed in gut microbial composition (*p* = 0.046) with lower microbial species richness (Chao1) (*p* = 0.032) and evenness (Shannon) (*p* = 0.0029). Fourteen of the twenty fecal amino acids including branch-chain- and aromatic- amino acids were increased among Hispanics (*q* < 0.05). Brain structural volumes in reward regions were decreased in Hispanics (pallidum, *q* = 0.036; brainstem, *q* = 0.011). Correlation patterns suggest complex brain-gut interactions differ by Hispanic ethnicity. In conclusion, Hispanics expressed a unique brain-gut microbial signature, which was associated with obesity despite sociodemographic and dietary differences. Addressing ethnic disparities guided by biologic phenotypes may unlock novel understanding of obesity heterogeneity and treatment strategies.

## 1. Introduction

Obesity is a heterogenous, chronic condition that has reached pandemic proportions over the past 50 years [1,2]. Defined as excessive fat accumulation, commonly diagnosed by a body mass index (BMI) ≥ 30 kg/m^2^, obesity has been associated with an increased risk of mortality, accounting for 19% of premature deaths, and is a major risk factor for other noncommunicable diseases including cardiovascular disease, diabetes mellitus, and cancer [3,4,5]. From 1975 to 2016, the global prevalence of obesity has nearly tripled from 3.2% to 11.1% in adult men and from 6.4% to 15.3% in adult women [6,7]. In the United States (US) alone, over 88 million adults (42.4%) are estimated to be obese, of which non-Hispanic blacks (49.6%) have the highest age-adjusted prevalence of obesity, followed by Hispanics (44.8%), non-Hispanic Whites (42.2%) and non-Hispanic Asians (17.4%) [8]. The heterogeneity in obesity prevalence between and within countries has been explained by not only ethnicity, but also socio-economic differences [9,10]. For instance, disparities in obesity prevalence between neighboring countries might be explained by exposure to obesogenic or a “Western-American” diet (high energy content, high sugar and fat, and low in fiber) [11]. Furthermore, prior studies that evaluate disparities in obesity prevalence in the US have found that factors such as cultural norms, poverty, indicators related to the food environment (i.e., access to supermarkets or fast food restaurants), gender, and other demographic groups are also associated with obesity outcomes [12]. Indeed, the obesogenic environmental and societal risk factors are multifaceted, and include dietary influences, social determinants, societal infrastructures, public health policies and beyond [13,14].

Current research supports the fundamental pathogenesis of obesity as an excessive energy intake overtime predisposed by genetic and epigenetic susceptibility and regulated by metabolic hemostasis [15,16]. Notably, studies have highlighted a key regulatory role of the brain-gut-microbiome (BGM) signaling in obesity development [1,2,4,5,6,17]. Research has shown that obesity is associated with phylum-level changes in the microbiota, reduced bacterial diversity and altered representation of bacterial genes and metabolic pathways [7]. Obesity-associated gut microbiomes has also been found to correlate with altered food metabolism through metagenomic and biochemical analyses, demonstrating that obese gut microbiomes have increased capacity to harvest energy from diet than lean gut microbiomes [18]. In particular, fecal metabolites, including branched chain amino acids (BCAA), aromatic amino acids (AAA), as well as their downstream metabolic byproducts, have been shown to influence glucose homeostasis and insulin resistance [19]. Signaling from the brain influences many gastrointestinal processes, including the gut microbiome [20]. Alterations in the brain’s key reward and emotional regulation regions may also contribute to dysregulation of appetitive behaviors and predisposition to obesity [21,22]. Conversely, signals from the gut microbiota can alter neural signaling to the brain [23,24]. Although the exact mechanisms of BGM axis remain incompletely understood, emerging evidence has suggested that gut microbiota output of amino acids in part influence neurodevelopmental processes including brain structural volume changes and contribute to the development of metabolic disorders [25,26]. The heterogeneity of obesity and its complex causes are now being increasingly recognized as reflected by a proposal to change in the International Code of Diseases (ICD) classification of obesity from “endocrine, nutritional and metabolic diseases” to an overarching parent category instead based on arrays of its multifaceted etiologies, degree of adiposity and health risks [27,28]. Albeit, significant questions remain about the relationships by which how in conjunction these biopsychosocial factors interact with BGM axis and contribute to obesity, and thus warrant further investigation.

With our aim to better understand the heterogeneity of obesity at the transitional junction of obesity development, we conducted a cross sectional study in healthy overweight and obese individuals to examine specific biopsychosocial interactions, such as the sociodemographic and dietary determinants of obesity and to identify potential BGM biomarkers of obesity. Studied variables included sociodemographic (age, gender, ethnicity, education, and annual income) and dietary-behavioral factors (dietary pattern). Gut microbiome, fecal amino acids, and brain structural volumes data were investigated as potential BGM biomarkers. Our study aimed to test the hypotheses that (1) interactions between covariates differed between overweight individuals compared to obese individuals, and (2) gut microbiome, fecal amino acids, and brain structural volumes would characterize differential biological phenotypes among the studied variables.

## 2. Materials and Methods

### 2.1. Study Participants

The study was comprised of 130 right-handed individuals, between the age of 18–60 years old without clinically significant medical or psychiatric conditions. Participants were recruited on consecutive basis at the University of California Los Angeles (UCLA) Center for Neurobiology of Stress and Resilience (CNSR) between July 2015 and August 2019, through advertisements posted in the UCLA and Los Angeles community. Medical and psychiatric conditions were screened using a standardized screening sheet and a physical exam by a trained registered nurse. All participants were classified as medically and mentally healthy after a clinical assessment that included a modified Mini-International Neuropsychiatric Interview Plus 5.0 [29]. Exclusion criteria included substance abuse, pregnancy, tobacco dependence (one-third pack or more daily), abdominal surgery, vascular risk factors, weight loss surgery, excessive exercise (more than 1 hour every day and marathon runners) or psychiatric illness. Although often associated with increased BMI, participants with hypertension, diabetes or metabolic syndrome were excluded to reduce heterogeneity. Also, participants with eating disorders, including digestive or eating disorders such as anorexia or bulimia nervosa were excluded for the same reason. Participants taking medications that interfere with the central nervous system or regular use of analgesic drugs were excluded. Participants were also excluded if they had been on antibiotics or probiotics within 3 months of recruitment.

All participants were in the overweight and obese category. In accordance to the World Health Organization (WHO) definition, BMI = 25–29.9 kg/m^2^ was defined as overweight, and ≥ 30 kg/m^2^ was obese. No participants exceeded 400 pounds (lbs) or BMI >39 due to magnetic resonance imaging (MRI) scanning weight limits. Written informed consent were obtained from all participants prior to surveys and data collection. All procedures complied with the principles of the Declaration of Helsinki and were approved by the Institutional Review Board at our institution (IRB # 16-000187).

### 2.2. Data Collection and Processing

#### 2.2.1. Anthropometrics Data

Our study included baseline data collection of participants’ sociodemographic information, body measurements and dietary consumption pattern. Sociodemographic data included information on age, gender, ethnicity, education, and annual income. We dichotomized age as “less than 30 years” (<30 yo) or “30 years or older” (≥30 yo), gender as “male” or “female,” ethnicity as “Hispanic” or “non-Hispanic,” education as “college graduate” or “non-college graduate,” and annual income as “less than $70 K” or “$70 K or more.” Body measurements collected include weight (kilograms) and height (centimeters). BMI was computed based on weight and height measurement and dichotomized as “overweight” (BMI = 25–29.9 kg/m^2^) or “obese” (BMI ≥ 30 kg/m^2^).

#### 2.2.2. Diet Habits

Dietary pattern was reported in a dietary consumption questionnaire, which asked participants to select the diet(s) that best reflect what they consume on a regular basis. Qualitative dietary patterns were reported as standard American diet or other diets, which we dichotomized as “American diet” or “non-American diet.” American diet was defined as diet characterized by high consumption of processed foods such as frozen and packaged foods as well as pasta and breads; meats, including red meat, fish, eggs, and dairy products were also consumed; vegetables and fruits were consumed but not in large quantiles. Other diets include Mediterranean, paleo, vegetarian, gluten free, dairy free, low FODMAP (fermentable oligo-, di-, monosaccharides and polys), or other restrictive diets. The diet questionnaire was validated and developed by the UCLA CNSR.

#### 2.2.3. Fecal Specimen

Fecal specimens were requested from eligible participants for microbiome and metabolite characterization. Participants were each provided with a fecal collection kit that included gloves, a collection hat, and an attached spoon. Fresh fecal specimens were stored in −80 °C freezer till sample processing. Fecal samples were aliquoted under liquid nitrogen.

### 2.3. Microbiome Characterization: 16S Ribosomal RNA Sequencing

Microbial DNA was extracted from fecal aliquots using the Powersoil kit as per the manufacturer’s instructions (MO BIO, Carlsbad, CA, USA). The V4 region of 16S ribosomal RNA (rRNA) genes was amplified and underwent paired end sequencing on an Illumina HiSeq 2500 (San Diego, CA, USA) [30]. The 253 base pair reads were processed using QIIME version 1.9.1 with default parameters [31]. Sequence depth ranged from 37,860 to 631,287 sequences per sample. Operational taxonomic units (OTUs) were identified using the May 2013 version of the Greengenes database, prefiltered at 97% identity. The OTUs were removed if they were present in fewer than 10% of samples. Alpha diversity (i.e., diversity within a sample) were calculated in QIIME using OTU-level data rarefied to 37,860 sequences. Beta diversity was calculated using root square Jensen-Shannon divergence distance, a phylogenetic metric that compares the fraction of a phylogenetic tree that is covered by the species present in one sample compared to another and visualized by principal coordinates analysis in R.

### 2.4. Fecal Amino Acids Characterization

Samples were processed by Metabolon on their global metabolomics and bioinformatics platform. Data were curated by mass spectroscopy using established protocols and software as previously described [32].

### 2.5. Brain Magnetic Resonance Imaging

#### 2.5.1. MRI Acquisition

Whole brain structural data were acquired using a 3.0 T Siemens Prisma MRI scanner (Siemens, Erlangen, Germany). Detailed information on the standardized acquisition protocols, quality control measures, and image preprocessing were previously published [33]. The following structural acquisition protocol was used: High resolution T1-weighted images were acquired: echo time/repetition time (TE/TR) = 3.26 ms/2200 ms, field of view (FOV) = 220 × 220 mm slice thickness = 1 mm, 176 slices, 256 × 256 voxel matrices, and voxel size = 0.86 × 0.86 × 1 mm.

#### 2.5.2. Quality Control and Preprocessing of Images

Structural images were included based on compliance with acquisition protocol, full brain coverage, minimal motion (Gibbs ringing), absence of flow/zipper, and minor atrophy/vascular degeneration. Preprocessing for quality control involved bias field correction, co-registration, motion correction, spatial normalization, tissue segmentation, Maximum relative motion thresholds for translation and rotation for each direction (x, y, and z) were set at 2 mm and 2°, respectively. No subjects presented with serious adverse imaging artifacts and no subjects exceeded motion thresholds.

#### 2.5.3. Structural Image Parcellation

T1-image segmentation and regional parcellation were conducted using FreeSurfer v.5.3.0 (Laboratory for Computational Neuroimaging Athinoula A. Martinos Center for Biomedical Imaging, Charlestown, MA, USA) following the nomenclature described in the Destrieux and Harvard-Oxford subcortical atlas. This parcellation results in the labeling of 165 regions, 74 bilateral cortical structures, seven subcortical structures, the midbrain, and the cerebellum.

#### 2.5.4. Brain Regions of Interest

Based on previous research, regions of interest were restricted to core regions of the reward and emotional regulation network (basal ganglia: caudate nucleus, globus pallidum, putamen, thalamus, nucleus accumbens, amygdala, hippocampus, and brainstem [including the substantia nigra/SN and ventral tegmental area/VTA]), as these regions have been implicated in brain-gut axis alterations associated with obesity [17].

### 2.6. Statistical Analysis

We conducted univariate and multivariate logistic regression models to estimate the unadjusted and adjusted odds ratios for covariates associated with the primary outcome, respectively. The primary outcome was overweight or obese categorization, for which we dichotomized individuals as “overweight” (BMI = 25–29.9 kg/m^2^) or “obese” (BMI ≥ 30 kg/m^2^). Multivariate models were performed by minimizing the Bayesian information criterion (BIC) using backward stepwise method. Subgroup multivariate analyses were performed on significant covariates. Model include all covariates (age, gender, ethnicity, education, annual income, and dietary pattern), against primary outcome of obesity status (overweight vs. obese). Descriptive statistics were calculated as frequencies and percentages. Statistical significance for logistic regression analyses was set at *p* < 0.05 and were conducted using JMP PRO (Mac, version 14.0, JMP, Cary, NC, USA). A sample size of 100 participants was estimated to test odds ratio of equality with the assumptions of obesity status proportional difference of 20% (60% vs. 40%) among studied covariates based on an alpha of 5% to provide adequate power of 80%.

Biological interactions including the gut microbiome, fecal amino acids and brain structural volumes were also examined against the primary outcome and covariates. Microbial data using 16S rRNA sequencing were analyzed for alpha diversity, beta diversity, and association of taxa abundance. Alpha diversity refers to metrics of diversity within a community (i.e., patient sample), which pertain to the total number of species (richness) or how evenly distributed the members of a community are among the species present (evenness) [34]. For our study, we used Chao1 (a metric of richness) and Shannon index (a metric of evenness) with 97% OTUs representing the equivalent of species. The significance of differences in alpha diversity was calculated by two-tailed t test. Beta diversity refers to comparison of microbial composition across communities (i.e., patient samples) based upon which species are present/absent or their relative abundances [35]. In our study beta diversity was calculated using root square Jensen-Shannon divergence distance and visualized by principal coordinates analysis in R. Adonis, a permutational ANOVA, was carried out using 10,000 permutations to test for differences in root-square Jensen distances across the various covariates. Association of microbial genera with color grade and significant covariates were evaluated using DESeq2 in R-studio, which uses an empirical Bayesian approach to shrink dispersion and fit non-rarified count data to a negative binomial model [36]. This method has previously been shown to be robust for detecting differences in the abundances of microbes in 16S rRNA datasets [37]. *p*-values for differential abundance were converted to *q* values to correct for multiple hypothesis testing (*q* < 0.05 for significance) [38]. Fecal amino acids and brain structural volumes were analyzed using multiple one-way ANOVA. Parameters were controlled and corrected for multiple hypothesis testing by false discovery rate (FDR). Finally, Pearson correlations (correlation coefficient = *r*) were performed with significant gut microbiome from DESeq2 analysis (*Actinobacteria, Bacteroidetes, Firmicutes, Fusobacteria, Proteobacteria*), fecal amino acids, brain structural volumes, sociodemographic continuous variables (age and BMI), and dichotomized dietary variable (“American diet” = 1, “non-American diet” = 0). Correlations were analyzed among but not within types and were separated by significant categorical covariates (e.g., “Hispanic” vs. “non-Hispanic”). *p*-values for correlation were converted to *q* values to adjust for covariates and correct for multiple hypothesis testing (<0.05 for significance). Significant correlations (*q* < 0.05) were used to build multi tripartite interaction networks for visualization. Continuous mapping displayed positive (red) or negative (blue) correlations with darker color representing stronger correlation.

## 3. Results

### 3.1. Baseline Participant Characteristics

Among the 130 studied participants, 62 (48%) were overweight and 68 (52%) were obese. About half of all participants were ≥30 yo (55%) and earned an annual income less than $70 K (56%). Majority of them were female (67%), non-Hispanics (60%), and non-college graduate (70%). Most consumed typical American diet (76%), while one quarter consumed a non-American diet (24%), which include Mediterranean, paleo, vegetarian, gluten free, diary free, low FODMAP, or other diets. Compared to the overweight participants, obese participants were more likely to be Hispanic (50% vs. 29%, *p* = 0.014), and to consume an American diet (84% vs. 68%, *p* = 0.031). Distribution of age, gender, education, and annual income were similar between the overweight and obese participants. Baseline characteristics are displayed in Table 1.

### 3.2. Odds of Obesity

In univariate analyses (Table 2), individuals were found to have higher odds of obesity if they were Hispanic [Odds Ratio (OR) 1.56, *p* = 0.014] or consumed an American diet (OR 1.57, *p* = 0.031). Controlling for all covariates, multivariate analyses (Table 2) found that individuals had higher odds of being obese if they were Hispanic [Adjusted OR (AOR) 1.56, *p* = 0.014].

### 3.3. Microbiome Analysis

**Figure 1:**
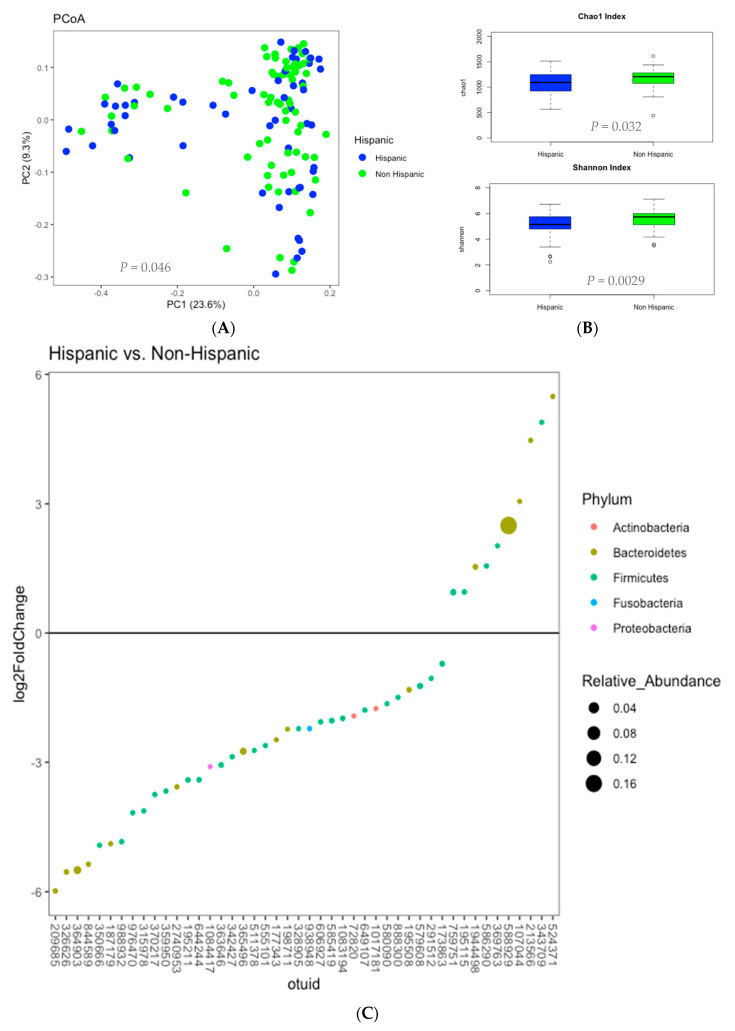
Microbial Profiles of Hispanic versus Non-Hispanic. (**A**). Beta Diversity. Principal coordinates analysis plots of microbial composition of Hispanic vs. Non-Hispanic. *p*-value is shown for the difference in root square Jensen-Shannon divergence distance matrix. (**B**). Alpha Diversity. Chao1 and Shannon Indexes illustrated in box plots of Hispanic vs. Non-Hispanic. (**C**). Differential expression of microbial genera (Phylum: (Genus)) associated with Hispanic vs. Non-Hispanic. Positive log2 fold change favor association to Hispanics in the following microbiome: *Bacteroidetes*: 107,044 (*Alistipes*), 524,371 (*Prevotella*), 1,944,498 (*Bacteroides*), 588,929 (*Prevotella*), 213,566 (*Bacteroides*). *Firmicutes*: 343,709 (*Ruminococcaceae*), 586,290 (*Lachnospiraceae*), 195,115 (N/A), 369,763 (*Coprobacillus*), 759,751 (N/A). Negative log2 fold change favor association to non-Hispanics in the following microbiome: *Actinobacteria*: 1,017,181 (*Rothia*), 72,820 (*Bifidobacterium*); *Bacteroidetes*: 364,903 (*Bacteroides*), 326,626 (*Bacteroides*), 209,685 (*Bacteroides*), 177,343 (*Bacteroides*), 198,711 (*Bacteroides*), 844,589 (N/A), 2,740,953 (*Bacteroides*), 187,179 (*Bacteroides*), 365,496 (*Bacteroides*), 195,508 (*Bacteroides*); *Firmicutes*: 342,427 (*Veillonella*), 585,419 (*Veillonella*), 1,083,194 (*Streptococcus*), 988,932 (N/A), 976,470 (N/A), 350,666 (N/A), 649,107 (N/A), 644,244 (N/A), 888,300 (*Streptococcus*), 579,608 (*Streptococcus*), 195,211 (N/A), 370,217 (N/A), 511,378 (*Veillonella*), 363,646 (*Ruminococcus*), 328,905 (*Oscillospira*), 606,927 (Clostridium), 315,978 (N/A), 359,950 (*Ruminococcus*), 555,101 (N/A), 173,863 (N/A), 291,512 (*Coprococcus*), 580,090 (N/A); *Fusobacteria*: 938,948 (*Fusobacterium*); *Proteobacteria*: 1,084,417 (*Lautropia*).

Based on the above associations of Hispanic ethnicity with obesity, we speculated that there may be alterations in the microbiome associated with Hispanic ethnicity which contribute to obesity. Microbial composition as represented by root square Jensen-Shannon divergence distance, a measure of phylogenetic similarity between samples, showed a statistically significant differences in the microbiome of samples of Hispanic participants compared to the non-Hispanic participants (univariate *p* = 0.046) (Figure 1A). Compared to the non-Hispanic, Hispanic participants had a significantly lower microbial species richness (Chao1) (*p* = 0.032) and evenness (Shannon) (*p* = 0.0029) (Figure 1B). Participants who were ≥30 yo also had a significantly lower microbial species richness (Chao1) (*p* = 0.036), and a trend toward a lower microbial species evenness (Shannon) (*p* = 0.099). Participants had similar beta and alpha diversity independent of other covariates (*p* > 0.05) or obesity status (obese or overweight), hence no additional statistical adjustment was performed.

Analysis of relative abundance of microbes at the phylum levels confirmed taxonomic shifts by Hispanic ethnicity (Figure 1C). Forty-six (46) significant operational taxonomic units (OTUs) comprised of five (5) microbial phyla were highlighted, including *Actinobacteria, Bacteroidetes, Firmicutes, Fusobacteria, Proteobacteria*. Among the significant OTUs, ten (10) of them have positive log2 fold change, favoring association with the Hispanic, while thirty-six (36) of them have negative log2 fold change, favoring association with the non-Hispanic. Of the ten (10) OTUs favoring association with the Hispanic, distinct phyla included *Bacteroidetes* and *Firmicutes*. Of thirty-six (36) OTUs favoring association with the non-Hispanic, all five (5) highlighted phyla were represented, including *Actinobacteria, Bacteroidetes, Firmicutes, Fusobacteria, Proteobacteria*.

### 3.4. Amino Acid Metabolites

Fourteen of the twenty fecal amino acids were increased among Hispanics (*q* < 0.05). These included BCAA (leucine, isoleucine, valine), AAA (phenylalanine, threonine, tryptophan), other essential amino acids (lysine, methionine), and certain non-essential amino acids (glycine, tyrosine, serine, alanine, aspartate, glutamine). Presence of fecal amino acids was not statistically different among other significant covariates including consumption of an American diet and obesity status. Amino acids analyses are displayed in Table 3.

### 3.5. Brain Structural Volumes

Regional brain structural volumes were decreased among Hispanics (pallidum, *q* = 0.036; brainstem, *q* = 0.011) and individuals who consumed an American diet (brainstem, *q* = 0.043). Compared to non-Hispanics, Hispanic participants also had a trend toward a decreased in thalamus volumes (*q* = 0.08). Hippocampus and amygdala structural volumes showed a trend towards association with obesity status (*q* = 0.16). The nucleus accumbens, caudate, and putamen structural volumes were not significantly different among any studied covariates or obesity status. Brain structural volumes analyses are displayed in Table 4.

### 3.6. Correlations Demonstrating Brain-Gut Microbiome Interactions

**Figure 2:**
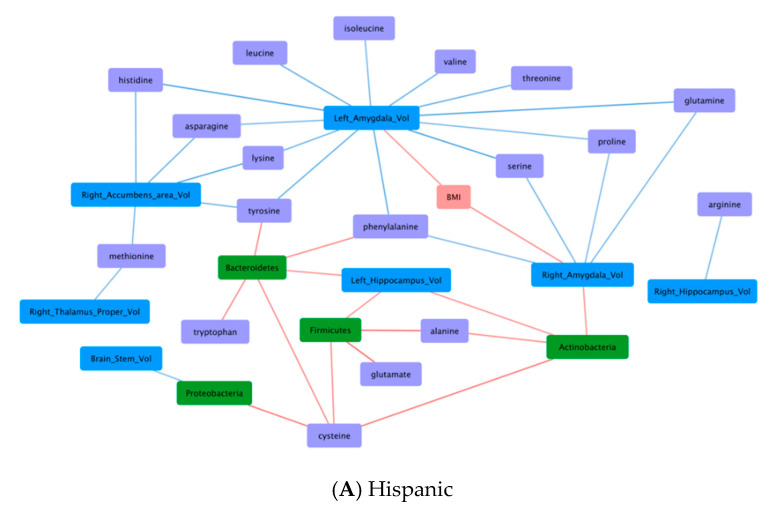

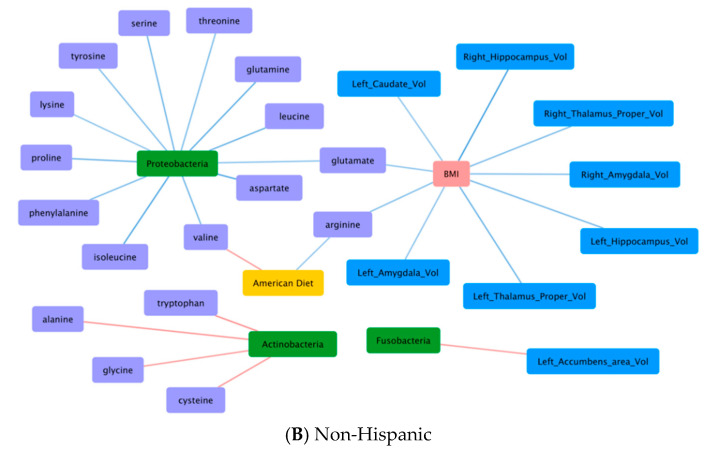
Pearson correlations of gut microbial, fecal amino acids, brain structural volumes, dietary pattern and BMI in Hispanics versus Non-Hispanics. Continuous mapping displayed positive (red) and negative (blue) correlation with darker color representing stronger correlation. Continuous variables include BMI (pink), diet (yellow), gut microbiome (green), fecal amino acids (purple), and brain structural volume (blue). Significant correlations (*q* < 0.05) are used to build interaction networks for visualization.

Differences in associations with BMI, dietary pattern, gut microbiome, fecal amino acids, and brain structural volumes were observed between the Hispanic and non-Hispanic participants (Figure 2) Six (6) notable patterns included: 1. BMI and brain structural volumes (BMI-Brain); 2. BMI and fecal amino acids (BMI-AA); 3. Gut microbiome and brain structural volumes (GM-Brain); 4. Gut microbiome and fecal amino acids (GM-AA); 5. Brain structural volumes and fecal amino acids (Brain-AA); and 6. Diet and fecal amino acids (Diet-AA). Among these patterns, Hispanic and non-Hispanic differed most distinctly with opposite correlations in BMI-Brain and GM-AA. In aggregate, correlation patterns suggest complex BGM interactions among the studied characteristics.

#### 3.6.1. BMI and Brain Structural Volumes (BMI-Brain)

In Hispanic, BMI was positively correlated to brain structural volume (Left amygdala: *r* = 0.28, *q* = 0.049; Right amygdala: *r* = 0.35, *q* = 0.049), whereas in non-Hispanic, BMI was negatively correlated to brain structural volumes (Left amygdala: *r* = −0.25, *q* = 0.045; R amygdala: *r* = −0.27, *q* = 0.037; Left thalamus: *r* = −0.32, *q* = 0.033; Right thalamus: *r* = −0.28, *q* = 0.037; Left caudate: *r* = −0.24, *q* = 0.049; Left hippocampus: *r* = −0.29, *q* = 0.036; Right hippocampus: *r* = −0.40, *q* = 0.017).

#### 3.6.2. BMI and Fecal Amino Acids (BMI-AA)

In Hispanics, BMI was not correlated to fecal amino acids; in non-Hispanics, BMI was negatively correlated to fecal amino acids (glutamate: *r* = −0.24, *q* = 0.045; arginine: *r* = −0.24, *q* = 0.037).

#### 3.6.3. Gut Microbiome and Brain Structural Volumes (GM-Brain)

With the exception of a negative correlation between *Proteobacteria* and brain stem seen in Hispanics (*r* = −0.28, *q* = 0.049), the gut microbiome was in general positively correlated to brain structural volumes among both Hispanic and non-Hispanic participants. In Hispanics, significant correlations included *Actinobacteria* to left hippocampus (*r* = 0.28, *q* = 0.049), *Actinobacteria* to right amygdala (*r* = 0.28, *q* = 0.049), *Bacteroidetes* to left hippocampus (*r* = 0.29, *q* = 0.049), and *Firmicutes* to left hippocampus (*r* = 0.28, *q* = 0.049). In non-Hispanics, significant correlations included *Fusobacteria* to nucleus accumbens (*r* = 0.25, *q* = 0.045).

#### 3.6.4. Gut Microbiome and Fecal Amino Acids (GM-AA)

In Hispanics, the gut microbiome was in general positively correlated to fecal amino acids. These include positive correlations of *Proteobacteria* to cysteine (*r* = 0.36, *q* = 0.044), *Bacteroidetes* to cysteine (*r* = 0.30, *q* = 0.049), tyrosine (*r* = 0.32, *q* = 0.049), tryptophan (*r* = 0.31, *q* = 0.049), phenylalanine (*r* = 0.31, *q* = 0.049), *Firmicutes* to cysteine (*r* = 0.30, *q* = 0.049), alanine (*r* = 0.40, *q* = 0.044), glutamate (*r* = 0.39, *q* = 0.044), and *Actinobacteria* to cysteine (*r* = 0.37, *q* = 0.044) and alanine (*r* = 0.30, *q* = 0.049). In contrast, non-Hispanics had mixed correlation patterns between gut microbiome and fecal amino acids. While *Actinobacteria* was still positively correlated to fecal amino acids [alanine (*r* = 0.28, *q* = 0.037); cysteine (*r* = 0.27, *q* = 0.039); glycine (*r* = 0.25, *q* = 0.045); tryptophan (*r* = 0.29, *q* = 0.036)], *Proteobacteria* was negatively correlated to fecal amino acids [glutamate (*r* = −0.25, *q* = 0.044); glutamine (*r* = −0.34, *q* = 0.030); threonine (*r* = −0.28, *q* = 0.037); aspartate (*r* = −0.38, *q* = 0.017); isoleucine (*r* = −0.37, *q* = 0.017); tyrosine (*r* = −0.27, *q* = 0.037); serine (*r* = −0.31, *q* = 0.035); proline (*r* = −0.31, *q* = 0.033); phenylalanine (*r* = −0.28, *q* = 0.037); leucine (*r* = −0.32, *q* = 0.033); lysine (*r* = −0.24, *q* = 0.046); valine (*r* = −0.30, *q* = 0.036)].

#### 3.6.5. Brain Structural Volumes and Fecal Amino Acids (Brain-AA)

In Hispanics, brain structural volumes were negatively correlated to fecal amino acids, whereas in non-Hispanics, brain structural volumes were not correlated to fecal amino acids. The significant correlations in Hispanics included Right thalamus to methionine (*r* = −0.29, *q* = 0.049); Right hippocampus to arginine (*r* = −0.31, *q* = 0.049); Left amygdala to serine (*r* = −0.40, *q* = 0.044), to threonine (*r* = −0.35, *q* = 0.049), to asparagine (*r* = −0.30, *q* = 0.049), to glutamine (*r* = −0.40, *q* = 0.044), to histidine (*r* = −0.40, *q* = 0.044), to lysine (*r* = −0.32, *q* = 0.049), to phenylalanine (*r* = −0.36, *q* = 0.049), to tyrosine (*r* = −0.34, *q* = 0.049), to leucine (*r* = −0.34, *q* = 0.049), to isoleucine (*r* = −0.31, *q* = 0.049), to valine (*r* = −0.33, *q* = 0.049), to proline (*r* = −0.30, *q* = 0.049); Right amygdala to serine (*r* = −0.31, *q* = 0.049), to glutamine (*r* = −0.30, *q* = 0.049), to phenylalanine (*r* = −0.29, *q* = 0.049), to proline (*r* = −0.29, *q* = 0.049); Right nucleus accumbens to asparagine (*r* = −0.32, *q* = 0.049), to histidine (*r* = −0.32, *q* = 0.049), to lysine (*r* = −0.38, *q* = 0.044), to tyrosine (*r* = −0.32, *q* = 0.049), and to methionine (*r* = −0.30, *q* = 0.049).

#### 3.6.6. Diet and Fecal Amino Acids (Diet-AA)

In Hispanics, dietary pattern was not correlated to fecal amino acids, whereas in non-Hispanics, American diet was positively correlated to valine (*r* = 0.25, *q* = 0.045) and negatively correlated to arginine (*r* = −0.24, *q* = 0.049).

## 4. Discussion

In this cross-sectional study of 130 healthy participants, Hispanics had decreased gut microbial diversity, increased fecal amino acids, and decreased volumes of pallidum and brain stem. Hispanics were more likely to be obese despite controlling for other sociodemographic and dietary differences. Correlation analysis suggests distinct alterations in BGM interactions in this population.

Our observation of the heighten obesity risk among Hispanics is supported by previous epidemiological studies [39,40,41]. According to the Center for Disease Control (CDC), Hispanic Americans are 1.2 to 1.8 times more likely to be obese than non-Hispanic whites across all age group [42]. This disproportional obesity prevalence contributes to significant health disparities among Hispanics [43,44,45,46,47]. Obesity-associated conditions such as heart disease, stroke, type 2 diabetes, and certain types of cancers have higher incidence among Hispanics [48,49]. Reasons for the disparity are likely multifaceted. Prior studies have found that Hispanics who are born in the US, have lived longer in the US, or arrive in the US at an early age have the highest obesity prevalence, suggesting that environmental factors such as acculturalization might influence obesity development [50,51,52,53,54,55]. Other obesogenic factors among Hispanics include higher rates of unemployment, higher levels of food insecurity, and poor access to healthcare resources [56,57,58,59]. Furthermore, biological factors, such as the gut microbiome, might also contribute to the heighten susceptibility of obesity among Hispanics [60,61,62,63]. A study comprised of 1674 Hispanic adults from the Hispanic Community Health Study/Study of Latinos (HCHS/SOL) found that those who relocated to the US at an earlier age had reduce microbial diversity and in turn higher risk of obesity [64].

We found distinct microbial profiles among Hispanics characterized by lower microbial diversity both in species richness (Chao1) and evenness (Shannon) [65,66]. Lower microbial diversity has been shown to be associated with chronic non-communicable diseases common in the developed world, such as inflammatory bowel diseases (IBD), colorectal cancer, diabetes, and obesity [67,68,69,70]. Low microbial diversity is characterized by proportional reduction of healthy microbes and predominance of facultative microbes, typically of the phyla *Firmicutes* and *Proteobacteria* [71]. The relative microbial abundance at the phylum levels presented in this study confirmed taxonomic shifts in the Hispanic participants, particularly distinct phyla of *Firmicutes* and *Bacteroidetes*. Several reports have shown a relative reduction in the phylum *Bacteroidetes* and a proportional increase in *Firmicutes* in obesity and metabolic syndrome [72,73,74]. Other studies, however, are contradictory, suggesting that microbiome influence on obesity development is more complex than a simple imbalance status of microbial phyla. These complex and often contradictory results based on gut microbial composition emphasize the importance of taking into account differences in microbial function [19,75,76,77,78]. For instance, studies have demonstrated that overweight and obese individuals have higher fecal short-chain fatty acids concentrations than their lean counterparts on a similar diet [79,80]. Other studies have found a marked increase in the serum concentrations of several essential amino acids including BCAA during high-fat diet induced obesity and glucose intolerance [81].

Our results demonstrate that Hispanics, when compared to non-Hispanics, had greater abundance of distinct fecal amino acids, including BCAA and AAA. Amino acids such as the BCAA and AAA are found to be more closely associated with metabolic disorders [82,83]. Alteration of plasma BCAA metabolism, for instance, was found to result in accumulation of toxic metabolites, which subsequently trigger mitochondrial dysfunction and stress signaling associated with insulin resistance [84,85]. Other studies have suggested that the gut microbiome alters the bioavailability and distribution of free amino acids in the gastrointestinal tract, and in turn modulates host metabolism [86]. Prospective investigations regarding the associations of ethnicity and fecal amino acids are few, particularly among Hispanics. Consistent with a global metabolomic signature associated with childhood obesity in the Hispanics, BCAA and their catabolites, propionylcarnitine and butyrylcarnitine, are significantly elevated in obese children [87]. Microbial metabolites can act both locally and systemically after being absorbed into the bloodstream. The impact of these biochemicals on human health is complex, and can potentially be both beneficial or toxic, depending upon the metabolite concentration or organ locality [88]. For instance, AAA degradation can yield a wide diversity of indolic and phenolic compounds that can act as toxins or neurotransmitters. Tryptamine, a byproduct of tryptophan catabolism, is a neurotransmitter that interacts with both indoleamine 2,3-dioxygenase and the aryl hydrocarbon receptor to heighten immune surveillance, and dampen the expression of pro-inflammatory cytokines, which have been implicated in the pathology of IBD and obesity [89,90,91,92]. Tryptamine can also both induce the release of or potentiate inhibitory response to serotonin, a neurotransmitter that is involved in metabolic processes including mood, appetite, and hemostasis [93,94]. Dysregulation of serotonin has been reported with conditions such as diabetes and obesity [95,96].

Amino acid differences were not observed in our study based on obesity status or dietary patterns. This is possibly due in part to insufficient statistical power from small sample size or to some extent from unmeasured confounders. For instance, similarity between metabolic states might be contributory. In other words, differences in fecal amino acid profiles might have been observed if study compared individuals who were obese (BMI ≥ 30 kg/m^2^) versus lean (BMI < 25 kg/m^2^) as opposed to overweight (BMI 25–29.9 kg/m^2^). Comparing to the lean participants, a study found that obese participants had a higher plasma concentration of amino acids including glutamate, tyrosine, alanine, proline, and BCAA [97]. Another study showed that plasma amino acids was associated with obesity based on the degree and indices of glucose intolerance [98]. Meanwhile, although studies have suggested that different dietary patterns induced differences in gut microbial composition and metabolites, findings have been inconsistent [99]. A study comparing individuals who were vegans and omnivores found that there were only few dietary group differences in gut microbial composition and no difference in fecal short chain fatty acids [100]. In another dietary experiment, a short 5-day change in diet (from baseline to either an animal- or a plant- based diet) found rapid and consistent shifts of microbial diversity compared with baseline [101]. These findings demonstrate that dietary-induced differences of gut microbiome and associated metabolites might be altered rapidly due to dietary adaptation, potentially confounding our studied results. Furthermore, it is worth noting that while fecal amino acids are derived from components of consumed diet, only some of fecal amino acid metabolites are produced by gut microbes. Dietary differences might not be detected due to sensitivity of our dietary questionnaire to capture detailed amino acid differences.

Integrating our findings with our correlation analysis, we found that association of gut microbiome to fecal amino acids (GM-AA) differed by Hispanic ethnicity. For instance, among non-Hispanics, *Proteobacteria* was negatively correlated to multiple fecal amino acids (glutamate, glutamine, aspartate, threonine, serine, lysine, proline, BCAA, AAA). In contrast among Hispanics, *Proteobacteria* was positively correlated to cysteine alone. *Proteobacteria* have been identified by some as a possible marker of microbiota instability and are often found to have increased abundance in chronic diseases such as inflammatory colitis [102]. In mice models, dominance of *Proteobacteria* was found to associate with proinflammatory state modulated by proinflammatory interleukins and reduction in immunoglobulin A [103]. Microbiome-mediated inflammatory processes have been found to associate with many chronic conditions including obesity [104]. Reports have also described that sulfur amino acids (e.g., cysteine) are independently associated with obesity and insulin resistance in Hispanics [105] In mouse model, supplementing cysteine decreases energy expenditure and promotes adiposity, whereas defects of cysteine-synthesizing enzymes decrease body weight [106]. In adipocytes, cysteine inhibits lipolysis and promotes lipogenesis via H2O2 production [107]. Another ethnic difference was that multiple positive GM-AA correlations were observed among Hispanics but were not present among non-Hispanics. Notably, these include relationships of microbiota (*Bacteroidetes*, *Firmicutes*, *Proteobacteria*) with fecal amino acids (glutamate, cysteine, tyrosine, alanine, and AAA) and downstream interactions to brain structural volumes (amygdala, hippocampus) and BMI, supporting the bidirectional interaction of BGM axis and potential role it plays on obesity development [108]. The exact mechanisms, however, of how these relationships contribute to the heighten obesity risk of Hispanics remained unclear and warrants future investigations.

We found distinct brain signatures to associate with Hispanics especially related to regions in the reward and emotion regulation network [109]. Prior studies have revealed that individuals who are obese exhibited smaller cortical thickness and total cerebral volume [110]. In a prospective observational study of 12,087 participants, total body fat (TBF) in men was negatively associated with all subcortical gray matter volumes (thalamus, caudate nucleus, putamen, globus pallidus, hippocampus, and nucleus accumbens, except for the amygdala), while TBF in women was negatively associated only with the globus pallidus volume [111]. Other studies have also found consistent patterns of obesity-related brain atrophy particularly in areas including the hippocampus, cingulate gyrus, and frontal lobes [112,113,114,115]. Literature supports that a reduction in neuronal fiber bundle length, which has been found to correlate with elevated BMI, is believed to contribute to the obesity-associated brain atrophy [116]. Reduction in structural volume of pallidum, which has been implicated in the rewarding effect of food, is correlated with heighten food seeking sensation among obese individuals [117,118]. Meanwhile, neurons in the brainstem are the input for neural circuit that integrates nutrient signals to control feeding behavior [119]. These studies demonstrating reduced morphometry in key reward and emotion regulation regions suggest that appetite and reward-related responses to food might be blunted among Hispanics, possibly contributing their heighten obesity risk. Although few studies have specifically investigated brain morphometry in ethnic groups with obesity, one study found that the volumes of key reward regions such as the globus pallidus and the brain stem were reduced among Hispanics with high BMI, highlighting the potential neurobehavorial associations to obesity [109].

Our correlation analyses support the inverse relationship of BMI and brain structural volumes (amygdala, thalamus, hippocampus, caudate) among non-Hispanics. In contrast, BMI was positively correlated to the amygdala among Hispanics. Amygdala is the emotion regulatory region of the brain, and is a key component of the corticostriatal circuit, comprising the orbitofrontal cortex, anterior cingulate cortex, insula and striatum. Studies have suggested that atrophy of the amygdala is linked to elevated impulsivity and addictive behaviors among adolescence and adults [120,121]. The inverse association between positive urgency (or tendency to act rashly under extreme positive emotions based on UPPS-P impulsive behavioral scale) and amygdala volume has also been shown in individuals with food addiction [122,123]. Furthermore, obesity influences multiple aspects of brain plasticity, including neurodevelopment, neurotrophins, neurogenesis, synaptogenesis, and ultimately activity at the brain network level [124]. Functional magnetic resonance imaging (fMRI) has found that high-calorie food cues activate brain reward regions including the amygdala and this increase activity was associated with abdominal fat and increased appetite in Hispanic women [125]. Stress exposure increases the release of amygdala neurotransmitters including glutamate, GABA, noradrenaline, and serotonin, activating a signal transduction molecular cascade that engages specific genes in neuroplasticity processes [126]. The alterations in levels of neuroactive metabolites can activate a pro-inflammatory milieu that has been implicated in the cross-link between energy metabolism, potentially contribute to the heighten obesogenic risk of Hispanics [20]. For instance, an animal study suggested that high fat diets trigger neurochemical changes through glutamatergic transmission (with upregulation of the glial glutamate carriers GLT-1 and GLAST), leading to a desensitization of NMDA receptors within the hippocampus (reduction of basal synaptic transmission and impairment of NMDA-induced long-term depression), which might account for reward signal deficits by triggering a downregulation of glutamate degrading enzymes [127]. Synaptic glutamate signaling in brain includes multiple interacting receptors, modulating co-transmitters and distinct regional dynamics that have been implicated in certain behavioral disorders, including food addiction [128,129].

### 4.1. Limitations

There are some limitations to our study. The cross-sectional study design limits our ability to establish temporal or causal relationships between covariates and outcome. Given the multifactorial nature of obesity, our covariates cannot possibly represent all relevant factors. Continuous (i.e., age, BMI) variables are dichotomized with the advantage for ease of statistic computation, but statistical power is expectantly compromised. Given multiple hypothesis testing, a larger sample size would be beneficial. Subject to recall or social desirability bias, self-reported dietary pattern can be strengthened with actual dietary consumption data with detailed recording of dietary amino acids consumption. Lastly, our participants are confined to Los Angeles community and our analyses are limited to healthy individuals who are overweight or obese, hence limiting generalizability.

### 4.2. Conclusions

Our findings draw attention to the influence of by biopsychosocial factors on obesity, including gut microbiome, fecal amino acids and regional brain structure. The study shows that Hispanics have a higher risk for obesity despite controlling for sociodemographic and dietary differences. Notably, Hispanics in this study express unique gut microbial, fecal amino acids, and brain structural volumes signatures that warrant future research. Microbial characterization in particular is an emerging predictive marker for therapeutics and might also serve as selection biomarker in obesity treatments and clinical trials. While still at its infancy, precision obesity care is undoubtably on the horizon [130]. By addressing ethnic disparities guided by precision phenotypes, we may potentially transform its impact on obesity care.

## Figures and Tables

**Table 1:** Baseline participant characteristics.

	Overall (*n* = 130)		Overweight (*n* = 62)		Obese (*n* = 68)		*p*
Characteristic	No	%	No	%	No	%	
Age							0.17
Less than 30 yo	59	45.4%	32	51.6%	27	39.7%	
30 yo or older	71	54.6%	30	48.4%	41	60.3%	
Gender							0.093
Female	87	66.9%	37	59.7%	50	73.5%	
Male	43	33.1%	25	40.3%	18	26.5%	
Ethnicity							0.014 *
Hispanic	52	40.0%	18	29.0%	34	50.0%	
Non-Hispanic	78	60.0%	44	71.0%	34	50.0%	
Education							0.51
College Graduate	37	29.8%	19	32.8%	18	27.3%	
Non-College Graduate	87	70.2%	39	67.2%	48	72.7%	
Annual Income							0.88
Less than $70 K	64	55.7%	31	56.4%	33	55.0%	
$70 K or More	51	44.3%	24	43.6%	27	45.0%	
Dietary Pattern							0.031 *
American Diet	99	76.2%	42	67.7%	57	83.8%	
Non-American Diet	31	23.8%	20	32.3%	11	16.2%	

* *p*-value < 0.05.

**Table 2:** Univariate and multivariate analyses of biopsychosocial and dietary characteristics associated with obesity.

.	Univariate Analyses			Multivariate Analyses		
Characteristic	Un-aOR	95% CI	*p* Value	aOR	95% CI	*p* Value
Age	0.79	0.55–1.12	0.17	–	–	
Less than 30 yo	–	–				
30 yo or older (reference)						
Gender						
Female	1.37	0.94–2.00	0.093	–	–	
Male (reference)	–	–				
Ethnicity						
Hispanic	1.56	1.08–2.26	0.014 *	1.56	1.08–2.26	0.014 *
Non-Hispanic (reference)	–	–		–	–	
Education						
College Graduate	0.88	0.59–1.30	0.51	–	–	
Non-College Graduate (reference)	–	–				
Annual Income						
Less than $70 K	0.97	0.67–1.42	0.88	–	–	
$70 K or More (reference)	–	–				
Dietary Pattern						
American Diet	1.57	1.02–2.41	0.031 *	–	–	
Non-American Diet (reference)	–	–				

* *p*-value < 0.05.

**Table 3:** Multiple one-way ANOVA *q* values of characteristics associated with fecal amino acids adjusted for FDR.

	Characteristics		
Fecal Amino Acids	Hispanic	American Diet	Obesity
Glycine	0.045 *	0.30	0.73
Serine	0.026 *	0.30	0.73
Threonine	0.030 *	0.30	0.73
Alanine	0.045 *	0.30	0.85
Aspartate	0.030 *	0.30	0.97
Asparagine	0.10	0.95	0.73
Glutamate	0.59	0.92	0.73
Glutamine	0.033 *	0.37	0.73
Histidine	0.15	0.30	0.97
Lysine	0.045 *	0.89	0.73
Phenylalanine	0.026 *	0.30	0.73
Tyrosine	0.030 *	0.30	0.73
Tryptophan	0.030 *	0.89	0.73
Leucine	0.030 *	0.30	0.73
Isoleucine	0.026 *	0.30	0.73
Valine	0.026 *	0.30	0.73
Methionine	0.026 *	0.30	0.85
Cysteine	0.99	0.30	0.73
Arginine	0.47	0.30	0.73
Proline	0.24	0.36	0.97

* *q* < 0.05. FDR: False discovery rate

**Table 4:** Multiple one-way ANOVA *q*-values of characteristics associated with brain structural volumes adjusted for FDR.

	Characteristics		
Brain Structures	Hispanic	American Diet	Obesity
Left Thalamus	0.08	0.52	0.22
Right Thalamus	0.22	0.47	0.24
Left Caudate	0.23	0.95	0.31
Right Caudate	0.23	0.95	0.31
Left Putamen	0.95	0.76	0.77
Right Putamen	0.95	0.76	0.84
Left Pallidum	0.036 *	0.88	0.99
Right Pallidum	0.036 *	0.68	0.99
Left Hippocampus	0.96	0.37	0.24
Right Hippocampus	0.96	0.74	0.16
Left Amygdala	0.83	0.98	0.85
Right Amygdala	0.83	0.98	0.85
Left Nucleus Accumbens	0.93	0.87	0.48
Right Nucleus Accumbens	0.93	0.87	0.48
Brain Stem	0.011 *	0.043 *	0.39

* *q*-value < 0.05. FDR: False discovery rate
